# Serological investigation of seven zoonotic pathogens in companion dogs in South Korea, 2018–2021

**DOI:** 10.1002/vms3.1380

**Published:** 2024-02-15

**Authors:** Yun‐Qi Song, Seol‐Ok Hong, Woo Bin Park, Suji Kim, Eun‐Seo Lee, Doo‐Sung Choen, Han Sang Yoo

**Affiliations:** ^1^ Department of Infectious Disease College of Veterinary Medicine Seoul National University Seoul Republic of Korea; ^2^ BK21 FOUR Future Veterinary Medicine Leading Education and Research Center Seoul National University Seoul Republic of Korea; ^3^ POSTBIO Co., Ltd Namyangju‐si Republic of Korea; ^4^ Research Institute for Veterinary Science Seoul National University Seoul Republic of Korea

**Keywords:** companion dog, One Health, serology, tick‐borne pathogen, zoonoses

## Abstract

Based on the current situation of Korean culture and society, the population of companion animals in South Korea is growing rapidly along with zoonotic risks. The current data regarding zoonotic infections in companion dogs reported in Korea is sparse. This study aims to investigate the seroprevalence of seven potential zoonotic pathogens in companion dogs in South Korea: *Anaplasma phagocytophilum*, *Borrelia burgdoferi*, *Ehrlichia canis*, *Coxiella burnetii*, *Brucella canis*, *Leptospira spp*. and canine influenza A virus. A total of 284 serum samples were collected from 2018 to 2021, and the immunoglobulin G (IgG) antibodies against 7 zoonotic pathogens were detected using enzyme‐linked immunosorbent assays. Samples were divided into five groups and analysed based on age. IgG antibodies against six of the seven pathogens were detected. The highest seropositivity rate was detected for canine influenza A virus exposure (59.1%) for which the rates were the highest in dogs under 1 year old and declined with age. Positivity rates of the other pathogens were relatively low: 1.76% for *Leptospira spp*., 1.40% for *A. phagocytophilum* and *E. canis*, 1.06% for *B. canis* and 0.35% for *B. burgdoferi*. No antibodies against *C. burnetii* were detected in this study. The exposure of dogs in South Korea to six zoonotic pathogens was serologically confirmed, highlighting a potential risk for human infection. The zoonotic risk of companion dogs cannot be neglected, and implementation of One Health approach should be advocated to establish effective preventive measures.

## Graphical abstract

This study aimed to investigate the seropositivity of seven zoonotic pathogens in companion dogs in Korea. Six pathogens were detected positive serologically. The results indicated that the potential zoonotic infection risks from companion dogs should not be neglected.

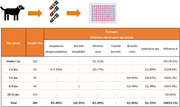

## 1 INTRODUCTION

Lifestyles in South Korea are changing, and along with this, companion animals have become a basic part of people's daily life. The number of families who are keeping companion dogs in Korea has increased dramatically over last few years (Cho et al., 2018). Along with this increase in companion animal numbers, the risk of zoonotic infections is inevitably increasing. Compared with cats, dogs are likely to present a higher risk because they are involved in more outdoor activities and tend to have closer contact with humans. This makes them more likely to come into contact with the zoonotic pathogens and to transfer them to humans. Among the zoonotic diseases associated with companion dogs, tick‐borne diseases are noteworthy. Many zoonotic pathogens can be transmitted from animals to humans by ticks, including *Anaplasma spp*., *Borrelia spp*., *Ehrlichia spp*. and *Coxiella burnetii* (Cui et al., 2017; Little et al., 2021).

*Anaplasma phagocytophilum* is a gram‐negative intracellular bacterium that can cause granulocytic anaplasmosis in humans and dogs (Kirtz et al., 2005). *Borrelia burgdorferi sensu lato spp*. is the causative agent of Lyme disease, and canine infections are an important part of its transmission (Steere et al., 2004). *Ehrlichia canis* is an intracellular bacterium that causes monocytic ehrlichiosis in dogs and humans (Rikihisa et al., 1992). *C. burnetii* is an intracellular bacterium infecting mammals and birds, and human infections resulting from contact with dogs have been reported previously (Shapiro et al., 2016).

In addition to tick‐borne diseases, other zoonotic diseases, including Brucellosis, Leptospirosis and canine influenza virus (CIV) infection, are noteworthy. Brucellosis is a zoonotic disease caused by *Brucella spp*., mainly *B. abortus* and *B. melitensis*. *Brucella canis* can also infect humans, although the infection risk is not high (Lucero et al., 2010; Wallach et al., 2004). *Leptospira spp*. have a wide host range and humans can be infected by direct contact with infected animals and/or their excretions. Major serotypes of CIV are H3N8 and H3N2, which were derived from equine and avian influenza viruses, respectively. Although no human infections with CIV have been reported, the high mutation rate of influenza A viruses makes them noteworthy pathogens.

Although zoonotic infections in companion dogs have been reported worldwide, few data regarding zoonoses have been reported from Korea. We investigated the serological prevalence of these seven zoonotic diseases in companion dogs in Korea to raise awareness of the zoonotic risks posed by companion animals. This scientific data may be helpful in the development of future control measures.

## 2 MATERIALS AND METHODS

### 2.1 Sampling

**FIGURE 1:**
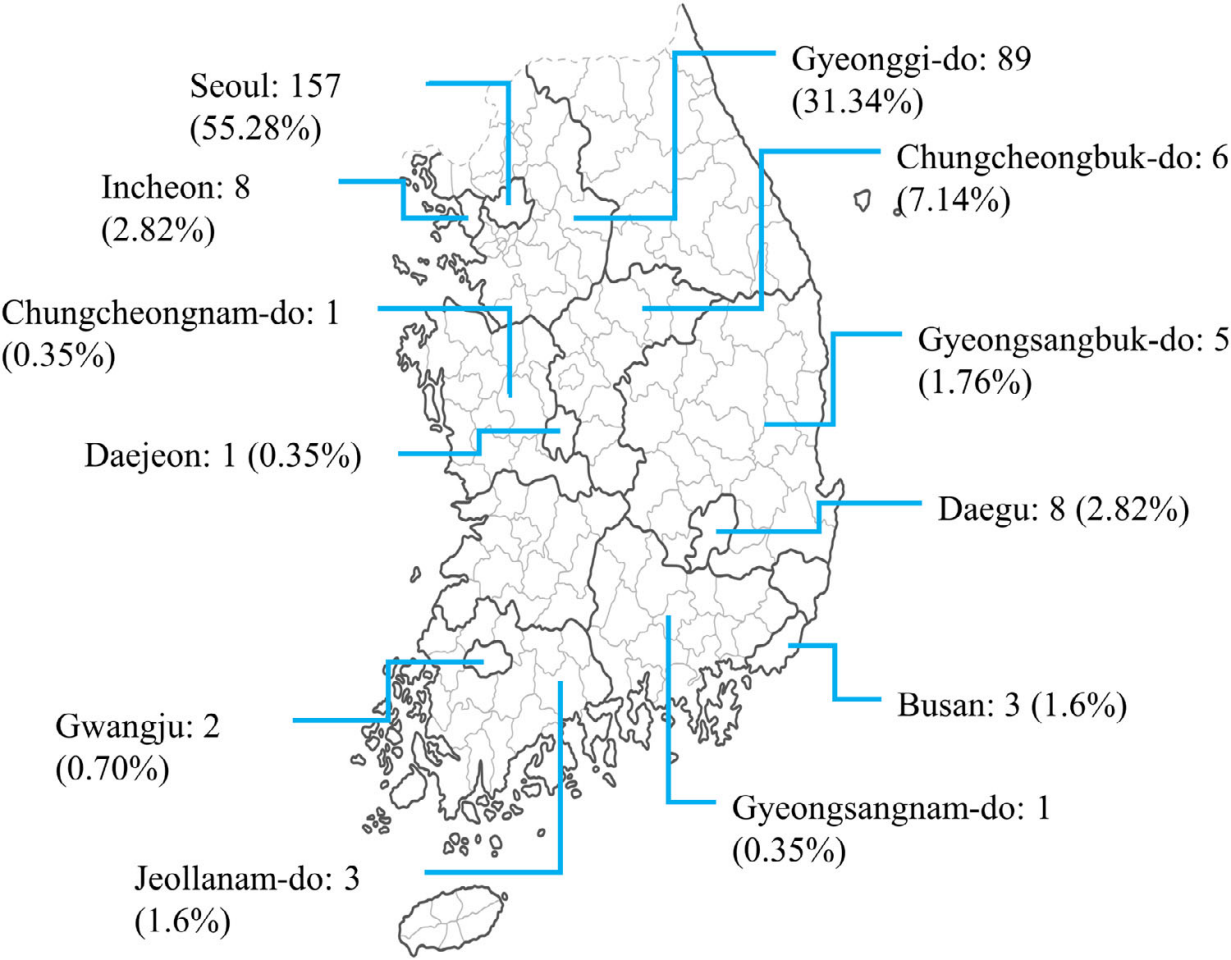
A map of companion dog serum sampling sites from different regions of South Korea: sample number in specific region (% of total sample number).

From 2018 to 2021, whole blood or serum samples were collected from 284 companion dogs. All recruited dogs were presented to animal clinics in several regions of South Korea (Figure 1, Table S1). Surplus blood samples were submitted and analysed at the same diagnostic laboratory. Age, breed and location of sampling were recorded (Table S2). The data were analysed based on the five different age groups: ≤1 year (*n* = 132), 1–2 years (*n* = 53), 3–5 years (*n* = 45), 6–9 years (*n* = 42) and 10–15 years (*n* = 12).

### 2.2 Serological investigation

Immunoglobulin G (IgG) against seven pathogens was detected using commercial enzyme‐linked immunosorbent assay kits shown in Table 1. The assays were conducted according to the manufacturer's instructions.

**TABLE 1:** Commercial enzyme‐linked immunosorbent assay (ELISA) kit used in the study.

Pathogen	ELISA kit product	Manufacturer
*Anaplasma phagocytophilum*	Anti‐Anaplasma phagocytophilum ELISA Dog (IgG)	Euroimmun
*Borrelia burgdorferi*	Anti‐Borrelia ELISA Dog (IgG)	Euroimmun
*Ehrlichia canis*	Anti‐Ehrlichia canis ELISA Dog (IgG)	Euroimmun
*Coxiella burnetii*	ID Screen Q Fever Indirect Multi‐species	IDVET
*Brucella canis*	VETLIS Brucella iELISA Canine kit	VETLIS
*Leptospira spp*.	Canine Leptospira IgG (IgG) Elisa Kit	AFG Bioscience
*Influenza A virus*	ID Screen Influenza A Antibody Competition Multi‐species	IDVET

Abbreviation: IgG, immunoglobulin G.

## 3 RESULTS

IgG antibodies directed against *A. phagocytophilum*, *Borrelia burgdoferi*, *E. canis*, *C. burnetii*, *B. canis*, *Leptospira spp*. and influenza A were detected (Table S2). The seroprevalence of Influenza A IgG was high (59.1%) with over half of the tested dogs having been exposed. The seroprevalence of other tested pathogens was low (<1.8%), and all 284 samples were negative for IgG antibodies directed against *C. burnetii*. One for *B. burgdoferi*, six for *B. canis*, five for *E. canis* and four for influenza A samples yielded borderline results, which for the purpose of the study, were considered to be negative. Classification of these borderline results as positive would double the seroprevalence of *B. burgdoferi* and *E. canis* and would triple that of *B. canis*; however, overall seroprevalence would nevertheless remain low (<3.2%). The seroprevalence of each pathogen is summarized in Table 2.

**TABLE 2:** Seroprevalence of seven pathogens categorised by age group.

		Pathogen positive results (seroprevalence)						
Age group	Sample size	*Anaplasma phagocytophilum*	*Borrelia burgdoferi*	*Ehrlichia canis*	*Coxiella burnetii*	*Brucella canis*	*Leptospira spp*.	Influenza A
Under 1 yr.	132	‐	‐	2 (1.51%)	‐	‐	‐	101 (76.5%)
1–2 yrs.	53	4 (7.54%)	‐	2 (3.77%)	‐	‐	1 (1.89%)	21 (39.6%)
3–5 yrs.	45	‐	‐	‐	‐	2 (4.44%)	3 (6.67%)	19 (42.2%)
6–9 yrs.	42	‐	1	‐	‐	1 (2.38%)	1 (2.38%)	20 (47.6%)
10–15 yrs.	12	‐	‐	‐	‐	‐	‐	7 (58.3%)
**Total**	**284**	**4 (1.40%)**	**1 (0.35%)**	**4 (1.40%)**	‐	**3 (1.06%)**	**5 (1.76%)**	**168 (59.1%)**

*Note*: ‐, No antibodies against the pathogen were detected.

Bold value indicates meaning of total to highlight.

## 4 DISCUSSION

The results of this study confirm that dogs in South Korea are exposed to zoonotic pathogens. A seroprevalence of 1.4% for *A. phagocytophilum* is significantly lower than the *Anaplasma spp*. infection rates of 13.8% (Lee et al., 2020) and 15.6% (Suh et al., 2017) reported in previous studies from Korea. As the detection methods used in the previous studies did not distinguish *A. phagocytophilum* from other *Anaplasma spp*., this result suggests that many *Anaplasma* infections in dogs may be caused by other *Anaplasma spp*. such as *Anaplasma platy*. Previously reported seroprevalence rates of *Anaplasma spp*. in China and America were 2.66% and 3.3%, respectively (Little et al., 2021; Zhang et al., 2017). Indeed, geographical differences in seroprevalence are not surprising as the prevalence of the vector will change according to region, climate and season, and populations will differ in number, lifestyle and management (Jaenson et al., 2012).

*Borrelia* antibodies were detected in only one of the 284 samples (0.3%), which is similar to the positivity rates documented in previous studies (Acharya & Park, 2021; Kim et al., 2020). Human *Borrelia* infection rates are high in America and Europe, but low in Asian countries (Koedel et al., 2015), and the same pattern has been observed in companion dogs (Little et al., 2021; Zhang et al., 2017). The results are also consistent with previous studies in Korea in which *B. burgdoferi* infection rates in companion dogs were lower than those of *A. phagocytophilum* and *E. canis* (Lee et al., 2020). The low prevalence of *B. burgdoferi* may be related to the predominant tick species in Korea (Kang et al., 2016).

IgG antibodies to *E. canis* were detected in only four dogs (1.40%), all of which were <2‐year old. In a previous study, the *E. canis* antibody‐positivity rate was 6.6% in companion dogs and 22.4% in military working dogs (Lee et al., 2020). Another study from Korea reported a seropositivity rate of 6.1% in hunting dogs (Lim et al., 2010). It may be that the dogs included in these studies were partaking in more frequent outdoor activities leading to a higher risk of tick exposure. Although *E. canis* has a worldwide distribution, regional infection patterns are extremely varied. For example, seroprevalence rates as high as 12.2% have been reported in Xinjiang, China (Mengfan et al., 2020) while a rate of 1.33% was reported in Eastern China (Zhang et al., 2017). Further afield, *E. canis* seroprevalence rates appear to be low in Japan (Inokuma et al., 2003), North America and the United States (Little et al., 2021) but reached 30.83% in Mexico (Movilla et al., 2016). These differences are likely to be explained by diversity of species and numbers of the regional tick populations, climate and environment. Notably, two individuals were concurrently seropositive for *A. phagocytophilum*. Co‐infection has been reported previously (Ebani, 2019; Movilla et al., 2016) and could be caused by tick co‐infection or by multiple ticks. Alternatively, it could arise due to cross‐reaction between the *A. phagocytophilum* and *E. canis* serological test methodology. In clinical situations, molecular detection can be helpful to confirm dual infection as well as to confirm active infection as opposed to previous exposure.

In this study, antibodies against *C. burnetii* were not detected in any of the 284 dogs. In the previously published canine studies, prevalence rates have been relatively low; however, as a previous study in South Korea found *C. burnetii* prevalence rates of 2.9%, the lack of seropositivity in our study was unexpected (Ebani, 2020; Lyoo et al., 2017). The possibility of false negative results cannot be excluded; however, as the manufacturer data confirm that the test has been validated for dogs, there is no reason to believe there was a particular problem with this specific test and seropositivity was generally found in dogs with predominantly outdoor lifestyles. Farm animals are the main reservoirs for *C. burnetii*, and seroprevalence rates are much higher in goats and cattle (Hwang et al., 2020; Jung et al., 2014). Hence, contact with livestock and their excretions is likely to be a major source of canine infection rather than tick bites. Accordingly, seropositivity is generally found in dogs with predominantly outdoor lifestyles (Ebani, 2020; Lyoo et al., 2017). Specific details regarding dog's environment and lifestyle were not recorded in this study, but a possible explanation for seronegativity would be a lack of exposure to farm animals in our particular study population.

Three dogs (1.06%) were seropositive to *B. canis* and were within the 3–9 years of age range. *B. canis* infection in dogs has been reported worldwide with reported seroprevalence rates ranging from 0.11% to 37.6% (Daly et al., 2020; Jamil et al., 2019; Weese et al., 2020; Whitten et al., 2019). Previous studies in Korea have reported similar positive rates to that found in this study; 0.9% and 2.5% in companion dogs and stray dogs, respectively (Jung et al., 2018). A Canadian study showing 100% infection in some kennels raised awareness of the zoonotic potential of *B. canis*, and another study in China strongly supported the zoonotic risks of *B. canis* in human brucellosis due to the rapid growth of the companion dog population (Yan et al., 2022).

Canine leptospirosis is a widespread disease that has been detected worldwide (Alton et al., 2009; Bertelloni et al., 2019; Major et al., 2014; Santos et al., 2021). In Asian countries, canine leptospirosis has been detected in China, Japan and Mongolia with seropositivity rates ranging from 7.3% to 29.3% (Koizumi et al., 2013; Odontsetseg et al., 2005; Shi et al., 2012). The seroprevalence rate of 1.76% found in this study was significantly lower than the 7.5% rate found in a previous study in Korea (Jung et al., 2008) and highlights the existence of regional differences. It is of concern that wild rodents and domestic animals are the main reservoirs for *Leptospira spp* (Adler, 2015), particularly as many infected animals remain asymptomatic. This means that contamination of the environment, including water and food supplies, can go undetected due to occult urinary and faecal bacterial shedding. This emphasizes the importance of rodent control in the prevention of leptospiral infection (Piredda et al., 2021).

Among the pathogens examined in this study, influenza A virus had the highest seropositivity (59.1%). Two types of CIV are emerging worldwide: The H3N8 strain, which is mainly endemic in the United States (Wasik et al., 2021), and the H3N2 CIV that has been endemic in Korea since 2004. As a result, CIV vaccination is widely promoted in South Korea. In this study, the highest seroprevalence was documented in very young dogs (<1year, 76.5%), presumably because most dogs are vaccinated against at CIV at a very young age. Another slight peak in seroprevalence was seen in the oldest dogs (10–15 years, 58.3%) the cause of which is unclear. It is possible that older dogs have had a greater chance of previous exposure. Molecular diagnostics would be helpful to further investigate CIV infection in these dogs, particularly in light of the large‐scale vaccination.

Despite low level prevalence rates for most of the infections studied, the results highlight the potential for infection transmission from companion dogs to humans. This emphasizes the importance of focusing not only on human infections but also considering that of their animal counterparts. The ‘One Health’ approach is an integrated approach that aims to balance and optimize the health of people, animals and the environment. Thus, as part of a wider network and information base, seroprevalence studies such as this one provide important information that can help to structure and consolidate preventive measures.

## 5 CONCLUSION

The exposure of companion dogs in South Korea to six zoonotic pathogens was serologically confirmed, highlighting a potential risk for human infection. The zoonotic risks of dogs cannot be neglected, and the One Health approach should be considered to establish effective preventive measure.

## AUTHOR CONTRIBUTIONS

*Data curation; formal analysis; methodology; validation; visualization; writing‐ original draft preparation; writing – review and editing*: Yun‐Qi Song. *Investigation; methodology; data curation; validation; writing – review and editing*: Seol‐Ok Hong. *Investigation; data curation; validation; writing review and editing*: Woo Bin Park. *Data curation; validation; writing review and editing*: Suji Kim. *Data curation; validation*: Eun‐Seo Lee. *Investigation; resources; data curation; writing review – and editing*: Doo‐Sung Choen. *Conceptualization; methodology; project administration; funding acquisition; resources; supervision; writing – review and editing*: Han Sang Yoo.

## CONFLICT OF INTEREST STATEMENT

The authors declare no conflicts of interest.

## ETHICS STATEMENT

The serum samples analysed in this study were collected from animal clinics by veterinarians and were approved by pet owners.

### PEER REVIEW

The peer review history for this article is available at https://publons.com/publon/10.1002/vms3.1380.

## Supporting information


**Table S1** Epidemiological information on the dogs by regions and species.

**Table S2** Seropositivity and information of individual dogs used in this study.

## Data Availability

Data available on request from the authors.
