# Supplementary material for: Serological investigation of seven zoonotic pathogens in companion dogs in South Korea, 2018–2021
Source: Vet Med Sci. 2024 Feb 15;10(2):e1380. doi: 10.1002/vms3.1380 (PMC10867870; doi:10.1002/vms3.1380)
Supplement: Supplementary file 1 — Table S1 Epidemiological information on the dogs by regions and species. [file VMS3-10-e1380-s001.docx]

Supplementary Table 1. Epidemiological information on the dogs by regions and species.

| \| Variable \|  \| No. tested \| No. (%) Positive by ELISA test \| \| \| \| \| \| \| \| \| \| \| \| \| \| \| --- \| --- \| --- \| --- \| --- \| --- \| --- \| --- \| --- \| --- \| --- \| --- \| --- \| --- \| --- \| --- \| --- \| \|  \|  \|  \| *Anaplasma phagocytophilum* \| \| \| *Borrelia burgdoferi* \| \| *Ehrlichia canis* \| *Coxiella burnetti* \| \| \| *Brucella canis* \| *Leptospira spp.* \| \| \| Influenza A \| \| region \| Seoul \| 157 \| 1 (0.64) \| \| / \| \| 2 (1.27) \| \| / \| 2 (1.27) \| \| \| \| 4 (1.75) \| \| 91 (57.9) \| \| Gyeonggi \| 87 \| 2 (2.30） \| \| 1 (1.15) \| \| 2 (2.3) \| \| / \| 1 (1.15) \| \| \| \| / \| \| 56 (64.3) \| \| Daegu \| 8 \| / \| \| / \| \| / \| \| / \| / \| \| \| \| 1 (12.5) \| \| 6 (75) \| \| Incheon \| 8 \| 1 (12.5) \| \| / \| \| / \| \| / \| / \| \| \| \| / \| \| 4 (50) \| \| Chungcheongbuk-do \| 6 \| / \| \| / \| \| / \| \| / \| / \| \| \| \| / \| \| 2 (33.3) \| \| Gyeongsangbuk-do \| 5 \| / \| \| / \| \| / \| \| / \| / \| \| \| \| / \| \| 3 (60) \| \| Busan \| 3 \| / \| \| / \| \| / \| \| / \| / \| \| \| \| / \| \| 2 (80) \| \| Jeollanam-do \| 3 \| / \| \| / \| \| / \| \| / \| / \| \| \| \| / \| \| 3 (100) \| \| Chungcheongnam-do \| 1 \| / \| \| / \| \| / \| \| / \| / \| \| \| \| / \| \| / \| \| Daejeon \| 1 \| / \| \| / \| \| / \| \| / \| / \| \| \| \| / \| \| / \| \| Gyeongsangnam-do \| 1 \| / \| \| / \| \| / \| \| / \| / \| \| \| \| / \| \| 1 (100) \| \| Gwangju \| 1 \| / \| \| / \| \| / \| \| / \| / \| \| \| \| / \| \| / \| \| age \| under 1 year \| 132 \| / \| \| / \| \| 2 (1.51) \| \| / \| / \| \| \| \| / \| \| 101 (76.5) \| \| 1-2 years \| 53 \| 4 (7.54) \| \| / \| \| 2 (3.77) \| \| / \| / \| \| \| \| \| 1 (1.89) \| 21 (39.6) \| \| 3-5 years \| 45 \| / \| \| / \| \| / \| \| / \| 2 (4.44) \| \| \| \| \| 3 (3.77) \| 19 (42.2) \| \| 6-9 years \| 42 \| / \| \| 1 (2.38) \| \| / \| \| / \| 1 (2.38) \| \| \| \| \| 1 (2.38) \| 20 (47.6) \| \| 10-15 years \| 12 \| / \| \| / \| \| / \| \| / \| / \| \| \| \| \| / \| 7 (58.3) \| \| breed \| Mix \| 45 \| 2 (4.44) \| \| / \| \| 1 (2.22) \| \| / \| 1 (2.22) \| \| \| \| \| 1 (2.22) \| 15 (33.3) \| \| Bichon frisé \| 34 \| / \| / \| \| \| / \| \| / \| / \| \| \| \| \| 1 (2.94) \| 19 (55.9) \| \| Poodle \| 34 \| / \| / \| \| \| / \| \| / \| / \| \| \| \| \| / \| 24 (70.6) \| \| Maltese \| 32 \| / \| / \| \| \| 1 (3.13) \| \| / \| / \| \| \| \| \| / \| 21 (65.6) \| \| Pomeranian \| 30 \| / \| / \| \| \| 1 (3.33) \| \| / \| / \| \| \| \| \| / \| 23 (76.7) \| \| Maltipoo \| 15 \| / \| / \| \| \| / \| \| / \| / \| \| \| \| \| / \| 8 (53.33) \| \| Shihtzu \| 9 \| / \| / \| \| \| / \| \| / \| / \| \| \| \| \| / \| 4 (44.4) \| \| Chihuahua \| 8 \| / \| / \| \| \| / \| \| / \| / \| \| \| \| \| 1 (12.5) \| 4 (50) \| \| Golden retriever \| 8 \| / \| \| / \| \| / \| \| / \| \| / \| \| \| \| / \| 5 (62.5) \| \| Welsh Corgi \| 7 \| / \| \| / \| \| / \| \| / \| \| / \| \| \| \| / \| 5 (71.4) \| \| Dachshund \| 6 \| / \| \| 1 (16.7) \| \| / \| \| / \| \| / \| \| \| \| / \| 3 (50) \| \| Shiba Inu \| 6 \| / \| \| / \| \| / \| \| / \| \| 1 16.7) \| \| \| \| / \| 4 (66.6) \| \| Border Collie \| 5 \| / \| \| / \| \| / \| \| / \| \| / \| \| \| \| / \| 4 (80) \| \| Coton De Tulear \| 5 \| / \| \| / \| \| / \| \| / \| \| / \| \| \| \| / \| 4 (80) \| \| French Bulldog \| 5 \| 1 (20) \| \| / \| \| 1 (20) \| \| / \| \| / \| \| \| \| / \| / \| \| Labrador Retriever \| 5 \| / \| \| / \| \| / \| \| / \| \| / \| \| \| \| / \| 4 (80) \| \| Yorkshire terrier \| 4 \| / \| \| / \| \| / \| \| / \| \| / \| \| \| \| / \| 4 (100) \| \| Italian Greyhound \| 3 \| / \| \| / \| \| / \| \| / \| \| / \| \| \| \| / \| 3 (100) \| \| Jindo \| 3 \| / \| \| / \| \| / \| \| / \| \| / \| \| \| \| / \| / \| \| Miniature Pinscher \| 3 \| / \| \| / \| \| / \| \| / \| \| / \| \| \| \| / \| 2 (66.6) \| \| Old English Bulldog \| 2 \| / \| \| / \| \| / \| \| / \| \| 1(50) \| \| \| \| / \| 1 (50) \| \| Samoyed \| 2 \| / \| \| / \| \| / \| \| / \| \| / \| \| \| \| / \| 2 (100) \| \| Shetland Sheepdog \| 2 \| / \| \| / \| \| / \| \| / \| \| / \| \| \| \| / \| 2 (100) \| \| Spitz \| 2 \| / \| \| / \| \| / \| \| / \| \| / \| \| \| \| / \| 2 (100) \| \| Afghan hound \| 1 \| / \| \| / \| \| / \| \| / \| \| / \| \| \| \| / \| / \| \| Akita \| 1 \| / \| \| / \| \| / \| \| / \| \| / \| \| \| \| / \| / \| \| Bedlington Terrier \| 1 \| / \| \| / \| \| / \| \| / \| \| / \| \| \| \| / \| / \| \| Boston Terrier \| 1 \| / \| \| / \| \| / \| \| / \| \| / \| \| \| \| / \| / \| \| Chow Chow \| 1 \| / \| \| / \| \| / \| \| / \| \| / \| \| \| \| / \| / \| \| Cocker Spaniels \| 1 \| / \| \| / \| \| / \| \| / \| \| / \| \| \| \| 1 (100) \| 1 (100) \| \| Sapsal \| 1 \| / \| \| / \| \| / \| \| / \| \| / \| \| \| \| 1 (100) \| 1 (100) \| \| Schnauzer \| 1 \| / \| \| / \| \| / \| \| / \| \| / \| \| \| \| / \| 1 (100) \| \| Siberian Husky \| 1 \| 1(100) \| \| / \| \| / \| \| / \| \| / \| \| \| \| / \| / \| |
| --- | --- | --- | --- | --- | --- | --- | --- | --- | --- | --- | --- | --- | --- | --- | --- | --- | --- | --- | --- | --- | --- | --- | --- | --- | --- | --- | --- | --- | --- | --- | --- | --- | --- | --- | --- | --- | --- | --- | --- | --- | --- | --- | --- | --- | --- | --- | --- | --- | --- | --- | --- | --- | --- | --- | --- | --- | --- | --- | --- | --- | --- | --- | --- | --- | --- | --- | --- | --- | --- | --- | --- | --- | --- | --- | --- | --- | --- | --- | --- | --- | --- | --- | --- | --- | --- | --- | --- | --- | --- | --- | --- | --- | --- | --- | --- | --- | --- | --- | --- | --- | --- | --- | --- | --- | --- | --- | --- | --- | --- | --- | --- | --- | --- | --- | --- | --- | --- | --- | --- | --- | --- | --- | --- | --- | --- | --- | --- | --- | --- | --- | --- | --- | --- | --- | --- | --- | --- | --- | --- | --- | --- | --- | --- | --- | --- | --- | --- | --- | --- | --- | --- | --- | --- | --- | --- | --- | --- | --- | --- | --- | --- | --- | --- | --- | --- | --- | --- | --- | --- | --- | --- | --- | --- | --- | --- | --- | --- | --- | --- | --- | --- | --- | --- | --- | --- | --- | --- | --- | --- | --- | --- | --- | --- | --- | --- | --- | --- | --- | --- | --- | --- | --- | --- | --- | --- | --- | --- | --- | --- | --- | --- | --- | --- | --- | --- | --- | --- | --- | --- | --- | --- | --- | --- | --- | --- | --- | --- | --- | --- | --- | --- | --- | --- | --- | --- | --- | --- | --- | --- | --- | --- | --- | --- | --- | --- | --- | --- | --- | --- | --- | --- | --- | --- | --- | --- | --- | --- | --- | --- | --- | --- | --- | --- | --- | --- | --- | --- | --- | --- | --- | --- | --- | --- | --- | --- | --- | --- | --- | --- | --- | --- | --- | --- | --- | --- | --- | --- | --- | --- | --- | --- | --- | --- | --- | --- | --- | --- | --- | --- | --- | --- | --- | --- | --- | --- | --- | --- | --- | --- | --- | --- | --- | --- | --- | --- | --- | --- | --- | --- | --- | --- | --- | --- | --- | --- | --- | --- | --- | --- | --- | --- | --- | --- | --- | --- | --- | --- | --- | --- | --- | --- | --- | --- | --- | --- | --- | --- | --- | --- | --- | --- | --- | --- | --- | --- | --- | --- | --- | --- | --- | --- | --- | --- | --- | --- | --- | --- | --- | --- | --- | --- | --- | --- | --- | --- | --- | --- | --- | --- | --- | --- | --- | --- | --- | --- | --- | --- | --- | --- | --- | --- | --- | --- | --- | --- | --- | --- | --- | --- | --- | --- | --- | --- | --- | --- | --- | --- | --- | --- | --- | --- | --- | --- | --- | --- | --- | --- | --- | --- | --- | --- | --- | --- | --- | --- | --- | --- | --- | --- | --- | --- | --- | --- | --- | --- | --- | --- | --- | --- | --- | --- | --- | --- | --- | --- | --- | --- | --- | --- | --- | --- | --- | --- | --- | --- | --- | --- | --- | --- | --- | --- | --- | --- | --- | --- | --- | --- | --- | --- | --- | --- | --- | --- | --- | --- | --- | --- | --- | --- | --- | --- | --- | --- | --- | --- | --- | --- | --- | --- | --- | --- | --- | --- | --- | --- | --- | --- | --- | --- | --- | --- | --- | --- | --- | --- | --- | --- | --- | --- | --- | --- | --- | --- | --- | --- | --- | --- | --- | --- | --- | --- | --- | --- | --- | --- | --- | --- | --- | --- | --- | --- | --- | --- | --- | --- | --- | --- | --- | --- | --- | --- | --- | --- | --- | --- | --- | --- | --- | --- | --- | --- | --- | --- | --- | --- | --- | --- | --- | --- | --- | --- | --- | --- | --- | --- | --- | --- | --- | --- | --- | --- | --- | --- | --- | --- | --- | --- | --- | --- | --- | --- | --- | --- | --- | --- | --- | --- | --- | --- | --- | --- | --- | --- | --- | --- | --- | --- | --- | --- | --- | --- | --- | --- | --- | --- | --- | --- | --- | --- | --- | --- | --- | --- | --- | --- | --- | --- | --- | --- | --- | --- | --- | --- | --- | --- | --- | --- | --- | --- | --- | --- | --- | --- | --- | --- | --- | --- | --- | --- | --- | --- | --- | --- | --- | --- | --- | --- | --- | --- | --- | --- | --- | --- | --- | --- | --- | --- | --- | --- | --- | --- | --- | --- | --- | --- | --- | --- | --- | --- | --- | --- | --- | --- | --- | --- | --- | --- | --- | --- | --- | --- | --- | --- | --- | --- | --- | --- | --- | --- | --- | --- | --- | --- | --- | --- | --- | --- | --- | --- | --- | --- | --- | --- | --- | --- | --- | --- | --- | --- | --- | --- | --- | --- | --- | --- | --- | --- | --- | --- | --- | --- | --- | --- | --- | --- | --- | --- | --- | --- | --- | --- | --- | --- | --- | --- | --- | --- | --- | --- | --- | --- | --- | --- | --- | --- | --- | --- | --- | --- | --- | --- | --- | --- | --- | --- | --- | --- | --- | --- | --- | --- | --- | --- | --- | --- | --- | --- | --- | --- | --- | --- | --- | --- | --- | --- | --- | --- | --- | --- | --- | --- | --- | --- | --- | --- | --- | --- | --- | --- | --- | --- | --- | --- | --- | --- | --- | --- | --- | --- | --- | --- | --- | --- | --- | --- | --- | --- | --- | --- | --- | --- | --- | --- | --- | --- | --- | --- | --- | --- | --- | --- | --- | --- | --- | --- | --- | --- | --- | --- | --- | --- | --- | --- | --- | --- | --- | --- |

/: no antibody was detected
